# Gravity-Driven Microfluidic Siphons: Fluidic Characterization and Application to Quantitative Immunoassays

**DOI:** 10.1021/acssensors.1c01524

**Published:** 2021-12-02

**Authors:** Nuno M. Reis, Sarah H. Needs, Sophie M. Jegouic, Kirandeep K. Gill, Sirintra Sirivisoot, Scott Howard, Jack Kempe, Shaan Bola, Kareem Al-Hakeem, Ian M. Jones, Tanapan Prommool, Prasit Luangaram, Panisadee Avirutnan, Chunya Puttikhunt, Alexander D. Edwards

**Affiliations:** † Department of Chemical Engineering and Centre for Biosensors, Biodevices and Bioelectronics (C3Bio), University of Bath, Claverton Down, Bath BA2 7AY, United Kingdom; ‡ Reading School of Pharmacy, University of Reading, Whiteknights Campus, Reading, RG6 6AD United Kingdom; § School of Biological Sciences, University of Reading, Whiteknights Campus, Reading, RG6 6AJ, United Kingdom; ∥ Dengue Hemorrhagic Fever Research Unit, Office for Research and Development, Faculty of Medicine Siriraj Hospital, Mahidol University, Bangkok, 10700, Thailand; ⊥ Molecular Biology of Dengue and Flaviviruses Research Team, Medical Molecular Biotechnology Research Group, National Center for Genetic Engineering and Biotechnology, National Science and Technology Development Agency, Pathum Thani, 73170, Thailand; # Siriraj Center of Research Excellence in Dengue and Emerging Pathogens, Faculty of Medicine Siriraj Hospital, Mahidol University, Bangkok, 10700, Thailand

**Keywords:** biosensor, microfluidic, siphon, smartphone diagnostics, dengue NS1, immunoassays, portable ELISA, porous membrane

## Abstract

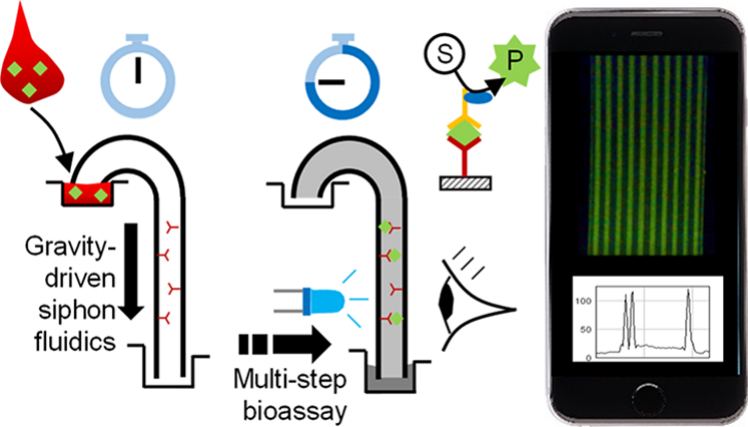

A range of biosensing techniques including immunoassays are routinely used for quantitation of analytes in biological samples and available in a range of formats, from centralized lab testing (e.g., microplate enzyme-linked immunosorbent assay (ELISA)) to automated point-of-care (POC) and lateral flow immunochromatographic tests. High analytical performance is intrinsically linked to the use of a sequence of reagent and washing steps, yet this is extremely challenging to deliver at the POC without a high level of fluidic control involving, e.g., automation, fluidic pumping, or manual fluid handling/pipetting. Here we introduce a microfluidic siphon concept that conceptualizes a multistep ″dipstick″ for quantitative, enzymatically amplified immunoassays using a strip of microporous or microbored material. We demonstrated that gravity-driven siphon flow can be realized in single-bore glass capillaries, a multibored microcapillary film, and a glass fiber porous membrane. In contrast to other POC devices proposed to date, the operation of the siphon is only dependent on the hydrostatic liquid pressure (gravity) and not capillary forces, and the unique stepwise approach to the delivery of the sample and immunoassay reagents results in zero dead volume in the device, no reagent overlap or carryover, and full start/stop fluid control. We demonstrated applications of a 10-bore microfluidic siphon as a portable ELISA system without compromised quantitative capabilities in two global diagnostic applications: (1) a four-plex sandwich ELISA for rapid smartphone dengue serotype identification by serotype-specific dengue virus NS1 antigen detection, relevant for acute dengue fever diagnosis, and (2) quantitation of anti-SARS-CoV-2 IgG and IgM titers in spiked serum samples. Diagnostic siphons provide the opportunity for high-performance immunoassay testing outside sophisticated laboratories, meeting the rapidly changing global clinical and public health needs.

The COVID-19 pandemic exposed a major gap in the point-of-care (POC) high-performance immunoassay detection of antigenic proteins, antibodies, and inflammatory and other important biomarkers, as explained in WHO’s coordinated global research roadmap.^1^ Laboratory immunoassays such as microplate enzyme-linked immunosorbent assay (ELISA) achieve analytical performance through multiple steps (reagent, washing, incubation, and enzymatic amplification). Lateral flow immunoassays (LFAs)^2^ perform immunochromatography through a heterogeneous porous nitrocellulose substrate, offering low cost and simplicity fulfilling all of WHO’s ASSURED^3^ requirements, but cannot match the analytical performance (sensitivity and quantitation) of multistep lab immunoassays such as microplate ELISA and are therefore not fit for many modern clinical needs. Multistep bioassays have been successfully automated yet rely on a ″wicking flow″ driven by capillary forces in substrates such as paper or nitrocellulose or a multilayer microfluidic device, resulting in uneven flow,^2^ limited control of incubation times, complex device design, and crossover of reagents, all compromising analytical performance. There remains a need for a portable multistep analytical approach or device able to deliver a sequence of immunoassay reagents and washes without access to fluid handling equipment (pipettors and pumps), approaching the usability of LFAs, and (ideally) capable of multiplexing a panel of measurements.

A range of power-free microfluidic^4−12^ devices using capillary action or capillary forces (unavoidable at small scales) to manipulate liquids have evolved from the LFA’s nitrocellulose membrane. These include paper-based capillary microfluidic and passive capillary microfluidic devices extensively reviewed elsewhere.^13^ Capillary phenomena^14^ have been combined with, e.g., electrochemical^15^ and electrostatic^16,17^ effects or more advanced designs^18^ to deliver features of fluid control essential for a multistep high-performance microfluidic immunoassay, including stop flow,^19^ prevention of bubble trapping,^20^ retention valves,^21,22^ and preprogrammed sequential delivery of reagents.^23−27^ However, the dependence on capillary forces constrains all these devices to the same limitations as rapid LFA tests, with the relationship between flow rate and flow resistance changing throughout the liquid wicking of the material. In some cases, a larger waste pad is readily used as a capillary ″pump″,^28^ governed by capillary forces and limited to small volumes of reagents and the sample. The flow rate is a key parameter as it governs the test time and, often, the analytical performance of the assay.^29,30^ As capillary forces are very sensitive to capillary diameter, small differences in porosity result in uneven superficial fluid velocities, which are characteristic of nitrocellulose membrane tests. Sequential fluid delivery, essential for autonomous multistep immunoassays, has been achieved through the use of, e.g., retention, burst or capillary valves (with fluids delivered according to their order of capillary pressure),^21^ matrix plugs (with sequential drainage of fluid based on the properties of the porous matrix),^25^ dissolvable barriers/vents (with the thickness of the hydrogel layer determining the sequence of drainage of each fluid),^27^ and dissolvable sugars (with the fluid sequence determined by the sugar concentration in each channel).^24^ Such strategies resulted in a significant overlap of reagents and can make the operation of devices challenging in terms of using small wash volumes or delivering good signal-to-noise ratios in enzyme-amplified immunoassays. With low analyte concentrations, even low residual levels of unbound enzyme/conjugate are sufficient to yield a significant background, compromising the analytical performance by raising the limit of detection. Microfluidic or microwell approaches fed manually into a ″funnel″ face similar challenges^31^ and require large wash volumes to remove all unbound enzyme from dead volume in the inlet well.

Microscale siphons have been mentioned in the literature for two very distinct applications: control of cell culture media in a tissue culture microwell/microchannel^32,33^ and control of fluid movement in centrifugal microfluidics.^34−36^ With centrifugal microfluidic devices, a ″siphon″ is incorporated horizontally and fluid flow is triggered by raising the centrifugal force to overcome the siphon crest (or neck). The system presented by Ozaki et al.^32^ for cell culture used pumped recirculation with fluid flow down a porous paper matrix driven by gravity, without overcoming a raised neck at higher hydraulic height and therefore not using the siphon effect. The microfluidic cell culture channel proposed by Jeong et al.^33^ required manual priming by a syringe and the cotton-yarn ″siphon″ pump operated at a hydraulic pressure lower than the cell culture feeding reservoir. Therefore, there are no previous reports of a vertical siphon being exploited to automate microfluidic multistep bioassays specifically enzyme-amplified immunoassays or ELISA.

We present a novel microfluidic siphon concept consisting of a strip of porous substrate (porous membrane, microcapillary, or microbored material) configured with a ″swan beak″ (see side view in Figure 1A). A homogeneous flow of sequential reagents is driven exclusively by hydrostatic pressure.^37,38^ The microfluidic siphon presents a set of distinctive features suitable for high-performance, quantitative, and multiplexed bioassays, including enzyme immunoassays/ELISA, differentiating from all power-free microfluidic approaches and sequential fluid delivery strategies previously proposed. We illustrated here the fluidic performance of a range of siphon devices, in particular, a multibore microcapillary film, single-bore glass microcapillaries, and a glass fiber porous matrix. In addition, we demonstrated the application as ELISA quantitation in patient samples using a highly sensitive fluorescence substrate, AttoPhos, cleaved by alkaline phosphatase (AP) to produce bright green fluorescence when an analyte is enzymatically detected (Figure S1).

**Figure 1:**
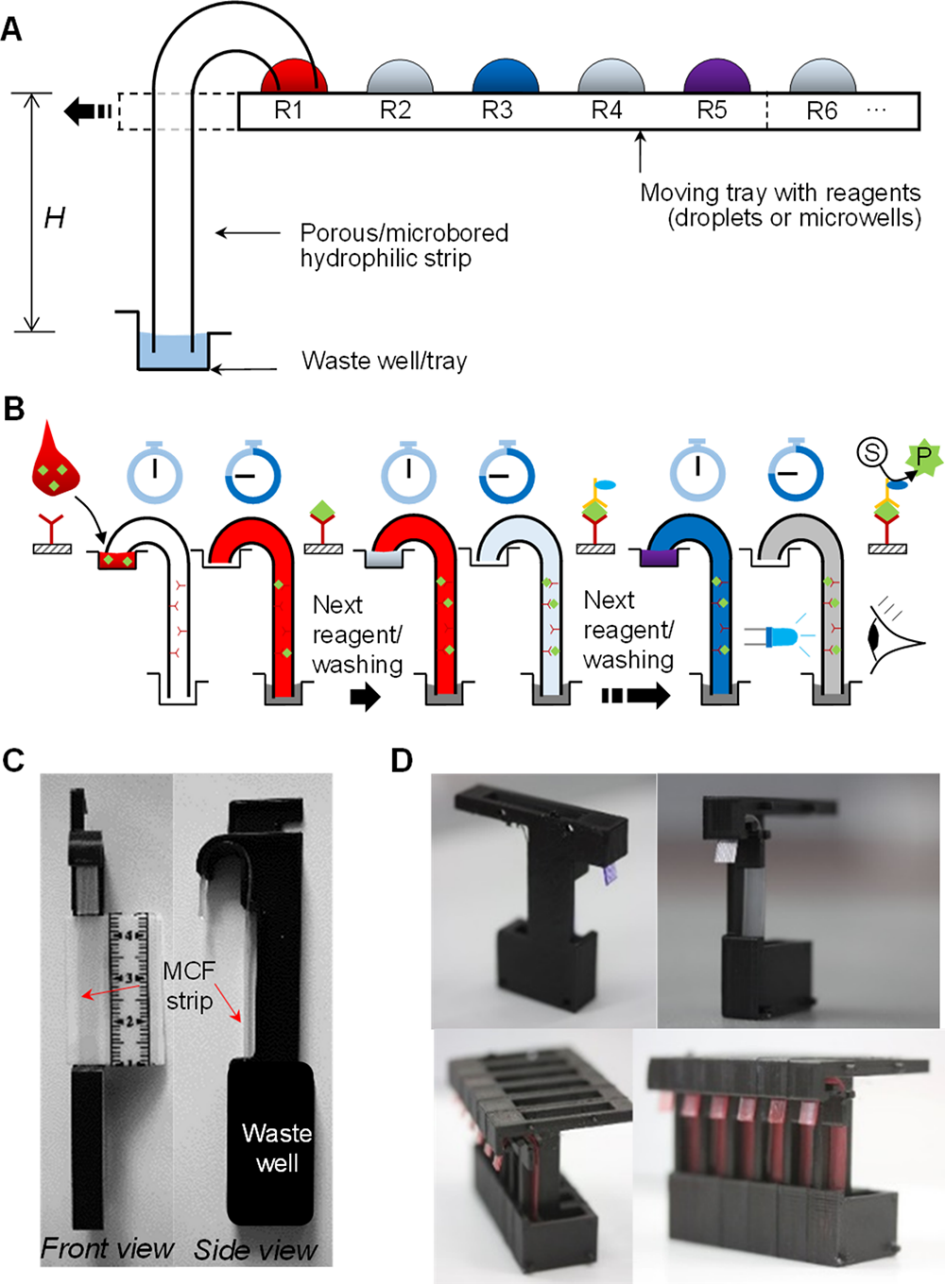
Operation of microfluidic siphons for multistep bioassays. (A) Liquid sample, reagents, and washing buffer (labeled R1–R6, shown in different colors) are automatically aspirated upon contact with the tip of the siphon with a hydrostatic liquid head, *H* (driving force for operation). The fluid goes through a neck at liquid high above the inlet before being discharged into a waste well/tray. (B) Microfluidic siphon is able to self-prime and allows start–stop operation with a sequence of reagents without emptying; insets show key steps for a sandwich ELISA with optical/fluorescence interrogation. (C) Photographs of a mass-manufacturable plastic siphon cassette that can be operated manually or using a robotic arm, loaded with an 89 mm long MCF strip and a large shallow waste reservoir (shown in side view), keeping *H* constant at 45 mm. (D) 3D printed cartridges loaded with a 47 mm long MCF strip (*H* = 25 mm) for single sample analysis (top) or stacked for up to 12 samples in parallel (bottom).

## Experimental Section

### Microcapillary Film (MCF) Strips

We used a 10-bore MCF, with a mean hydraulic diameter (*d*_c_) of 204 ± 13.8 μm, mass-manufactured from Teflon FEP material through a melt-extrusion process,^39^ with a cross section and *d*_c_ for each individual microcapillary shown in Figure S3A,B, respectively. The procedures for coating the strips with a high-molecular polyvinyl alcohol (PVOH) solution, design, and operation of MCF-based stacking siphons are fully described in the Supporting Information.

### Glass Capillary Siphons

Hydrophilic, 142.4 mm long single-bore circular glass capillaries with measured OD 1.0 mm and ID 578 μm sourced from World Precision Instruments (Sarasota, Florida, US). Capillary siphons were produced having a total length, *L* constant at 142.4 mm and net hydrostatic liquid height, *H* (Figure 1A) and *H*/*L* ratio varied by bending the glass capillary at different points along the length with a Bunsen burner.

### Glass Fiber Porous Matrix Siphons

A grade 8964 lateral flow conjugate pad (Ahlstrom, Pont-Evêque, France) was cut into 7 mm wide and 80 mm long strips, and ends were placed in microwells with the inlet 50 mm (vertical) higher than the outlet, i.e., *H* = 50 mm.

### Dengue ELISA NS1 Viral Antigen Quantitation and Serotype Identification

MCF strips (47 mm long) were prepared as follows: a 1.4 m length of MCF was coated with a panel of serotype-specific capture antibodies against NS1 produced in-house (Table S1) at 60 μg/mL for DENV1 and 40 μg/mL for DENV2–4 in duplicate capillaries; these concentrations were selected as sufficient to yield a stable half-monolayer of antibody on the surface of Teflon FEP microcapillaries as characterized previously.^40^ A positive control capillary was coated with 1 μg/mL recombinant DENV2 NS1, and a negative capillary was filled with PBS alone. These multiplexed MCF strips were incubated at RT for 1 h, and the antibody solution replaced with 0.1 mg/mL PVOH (MW 146,000–186,000 g/mol, Sigma Aldrich, UK); the strips were coated for a further 2 h and finally blocked by filling up with 4% w/v BSA (GE Healthcare Life Sciences, UK) and incubated overnight at 4 °C before cutting into 47 mm test strips that were fitted into siphon holders. All assay steps used a 60 μL reagent, sample, or wash volume; unless otherwise stated, wash steps were left for 30 s to allow the full discharge of the wash volume. Samples were either neat patient plasma or uninfected control plasma spiked with recombinant DENV NS1. Neat plasma was incubated for 10 min followed by wash with 0.1% w/v Tween 20 (Sigma Aldrich, Dorset UK) in PBS (PBS-T). Biotinylated pan-serotype DENV NS1 antibody manufactured in-house was added at 10 μg/mL, diluted in 4% BSA and 10% rat serum (Sigma Aldrich, UK), and incubated for 5 min followed by a PBS-T wash. Streptavidin-AP conjugate (Sigma Aldrich, Dorset, UK) at 1:1000 dilution in 4% BSA was incubated for 5 min followed by three sequential washes with PBS-T and then addition of the fluorescence substrate.

### Anti-SARS-CoV2 ELISA Antiviral Titer Antibody Quantitation

MCF test strips were coated in all 10 capillaries with 40 μg/mL of SARS-CoV-2 S1 protein produced in-house by incubating for 1 h at RT. The antigen solution was replaced with PVOH 0.1 mg/mL for 2 h at RT and then a blocking buffer (Superblock + 10% FBS + 5% GS) and subsequently stored at 4 °C in the blocking buffer prior to cutting 47 mm long test strips fitted into siphon holders. The seroreactivity against the ″S1″ antigen was measured using a conventional endpoint titer protocol, with plasma derived from whole blood volunteer samples stabilized in 4% sodium citrate (Sigma Aldrich, UK) serially diluted in the Seablock blocking buffer (ThermoFisher Scientific, UK). The patient samples were incubated for 10–20 min in the siphon devices followed by three washes with PBS-T totaling 5 min. The secondary antibody, anti-human IgG conjugated with alkaline phosphatase (ThermoFisher Scientific UK) at 1 μg/mL in the Seablock blocking buffer, was incubated for a total of 10–20 min before being washed three times with PBS-T allowing a 5 min total wash duration and then addition of fluorescence substrate.

### Imaging of the Fluorescent Immunoassay Siphon Strips

For imaging both SARS-CoV-2 and DENV immunoassay strips, the AttoPhos AP Fluorescent Substrate System (S1000, Promega, UK) was added as the final substrate to the siphons and the fluorescent image was captured using both a Canon S120 digital camera and an iPhone 6S smartphone after 15 min incubation for DENV immunoassay or 5 min for SARS-CoV-2 immunoassay. Samples were illuminated using an open-source blue LED transilluminator, and images were taken through an amber acrylic emission filter (IOrodeo, Pasadena, CA, USA) with 12–16 test strips imaged simultaneously in a single image alongside the reference strip, representing 160 measurements per photo. The fluorescence signal for each individual capillary was determined from the peak green channel pixel intensity using Image J (NIH, USA). The relative fluorescent intensity was calculated relative to a reference MCF strip loaded with 5 μM (DENV NS1) or 2 μM (anti-SARS-CoV2) fluorescein to normalize fluorescence signal as reported previously.^37,38^

### Anti-SARS-CoV2 ELISA Titer Antibody Quantitation in Microtiter Plates

For the COVID-19 assay in microtiter plates, 10 μg/mL of SARS-CoV2 S1 antigen diluted in carbonate buffer (Sigma-Aldrich, UK) was coated onto the plate (Nunc Immunosorp) in duplicates and incubated overnight at 4 °C. The plate was washed three times with PBS-Tween 20 and blocked three times with Superblock. Patient samples were diluted as described above and incubated for 1 h at RT. Following four washes with PBS-Tween 20, the secondary antibody was diluted as described above and incubated for 1 h at RT. Finally, following four washes with PBS-T, the chromogenic substrate 3,3′,5,5′-tetramethylbenzidine (TMB) (Europa Bioproducts, UK) was added and color development was stopped by the addition of an equal volume of 0.25 M sulfuric acid. Absorbance was read at 440 nm against a reference read at 700 nm. For each set of duplicates, the average and the standard deviation were calculated.

### Simulated Anti-SARS-CoV2 ELISA Titer Antibody Quantitation

Similarly to the COVID-19 assay in MCF, various levels of recombinant human IgG and IgM anti-COVID-19 [CR3022] antibodies (Absolute Antibodies, UK) were spiked into the control human serum. The recombinant antibodies were spiked into the prepandemic human serum diluted 1:20 in Seablock at 50 μg/mL (high IgG/M) or 5 μg/mL (low IgG/M). A ″no IgG/M″ control consisted of the control prepandemic human serum diluted 1:20 in Seablock only. As previously, the samples were serially diluted in the Seablock blocking buffer before being incubated in the MCF strips.

## Results and Discussion

### Microfluidic Siphon Concept

We have delivered the vision of a pumping-free, microfluidic siphon able to sequentially aspirate a sample plus multiple reagents, separated by incubation steps and washings, from a preloaded reagent tray or microwell strip, located at a vertical, *z*-position (i.e., also called hydrostatic head) higher than the receiving waste well, as depicted in side view diagrams in Figure 1A,B. The microfluidic siphons require a very minor level of intervention from the operator in moving/depositing reagents in trays, which can be done by an unskilled person in contrast to, e.g., a lab-based ELISA. The full procedure for the operation of a four-channel MCF siphon cassette is shown in the film footages provided in the Supporting Information. As the flow is entirely governed by the net hydrostatic liquid head (*H*), the siphon can virtually operate continuously with any porous (hydrophobic and hydrophilic) material of any length *L* (we have successfully tested siphons with *H* and *L* values as large as 1 m; results are not shown). However, it is essential that the porous strip is hydrophilic to self-prime the siphon and initiate flow without input from the operator, just as with a capillaric microfluidic device. This first priming stage is essential to raise the liquid height of the sample or first reagent from the tray up and over the neck of the siphon, which happens by capillary action.

We incorporated the siphon in mass-manufacturable plastic cassettes similar to LFA cases (Figure 1C,D). In contrast to LFAs and capillaric circuits, the microfluidic siphon operates vertically, being solely powered by gravity once the first liquid overcomes the neck. Note that for devices operated through capillary action including LFAs, there is no interest in operating those vertically as capillary forces are quickly offset by fluid resistance and gravity, impairing the performance of the device. The height of the siphon neck is minimized to reduce material waste and flow resistance but must be sufficient to be immersed into the well or droplets in the reagent tray. The neck height should also not exceed the equilibrium height given the Laplace pressure of the hydrophilic channel, which for polyvinyl alcohol (PVOH) coated 10-bore, Teflon FEP melt-extruded MicroCapillary Film (MCF) strips^41^ used here we measured as 58 ± 3.0 mm (Supporting Information, Table S2 and Figure S3).

### Fluidic Measurement

**Figure 2:**
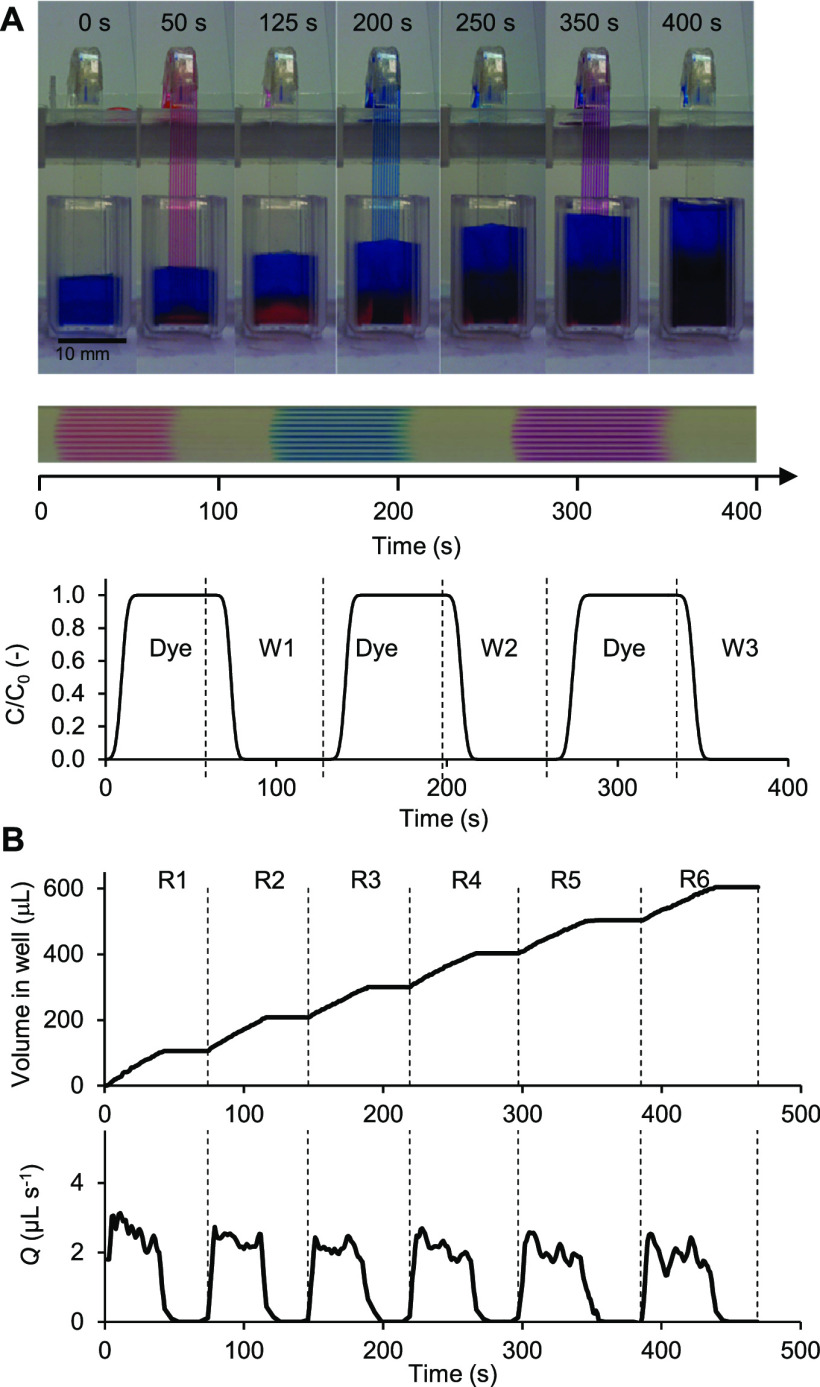
Fluidic operation of 10-bore microfluidic siphons. (A) Sequential flow of reagents from 100 μL droplets through a 10-bore MCF siphon (*H* = 30 mm, *L* = 60 mm) into a transparent 1 mL plastic cuvette preloaded with 300 μL of buffer stained with blue food dye (to facilitate image analysis and processing). A transparent washing buffer droplet approaches (0 s) the siphon inlet, then the tray is moved to a dark pink droplet (50 s), and the procedure was repeated for five further steps. The horizontal montage (middle) shows an RGB time sequence of the siphon imaged at 20 mm from the outlet of the strip, with the sequence of liquid ″reagents″ passing through the 10 individual parallel microcapillaries along time. Breakthrough curves (bottom) with the respective normalized concentration (*C*/*C*_0_) of dye and washing showing that the flow of reagents is very reproducible. Note that the total time shown for each reagent in the *x* axis corresponds to the time taken to fully discharge the volume of the droplet plus the time allowed for stop-flow ″incubation″ on each step. The siphon shown was designed with a linear (axial) neck distance of 30.0 mm curved at 180° with a diameter of curvature equal to ∼19.1 mm. (B) Liquid volume discharged through a 10-bore MCF siphon (*H* = 40 mm, *L* = 70 mm) measured over time (top) by imaging the volume in the waste cuvette, and the first derivative (bottom) yielding the instantaneous discharge flow rate (*Q*) plotted against time. The vertical dashed lines represent the time at which a fresh 100 μL droplet stained with 0.05% (w/w) indigo carmine was moved to interface the inlet of the siphon (a total of 75 s allowed for each reagent/washing).

Figure 2A shows the sequential discharge of the dye and buffer from a plastic tray (top image) through a siphon using MCF strips with *L* = 60 mm.^41^ A collage (Figure 2A bottom) illustrates the dye color at the strip center over time, confirming the distinct sequential delivery of the dye and wash, with no detectable fluid overlap. Washing efficiency was quantified by breakthrough curves with a dye (Figure 2A middle and Supporting Information) showing siphon operation with only 100 μL of fluid, with normalized concentration rapidly rising from 0 to 1 (for dye) and falling from 1 to 0 (washing). Further imaging of colored and fluorescent dye reagents using the siphon cassette shown in Figure 1C confirmed that reagent delivery can be automated (see Supplementary Methods in the Supporting Information). The dye–wash sequences in Figure S2 show that reagents are rapidly equilibrated in individual microcapillaries through simple molecular diffusion.

The microfluidic siphon delivered a constant discharge flow rate throughout >90% of the reagent’s volume (Figure 2B), only reducing at the start and end due to fluid acceleration upon contact with the liquid reagent, and deceleration as the reagent volume is exhausted. As a reference, for the devices mentioned in Figure 2 (10-bore), the siphon yielded a flow rate of ∼2.5 μL/s, taking ∼40 s to fully discharge 100 μL of fluid.

As flow in the siphon stops between each reagent or wash, the fluid is fully retained within the membrane or microcapillary by the inlet Laplace pressure, without emptying, allowing self-controlled stop-flow incubation with the sample, reagent, or wash. Tests with siphons of varying *L* revealed that the liquid is retained in the microcapillaries or microporous membrane even for values of *H* greater than the maximum measured equilibrium capillary rise^41^ (see Table S2 and Figure S3). In practice, the cost of the device is reduced with small *L*; therefore, minimizing *H* is desirable. For incubation times of 5–30 min (typical of microfluidic immunoassays^39^), no evaporation or air bubble entry was detected.

**Figure 3:**
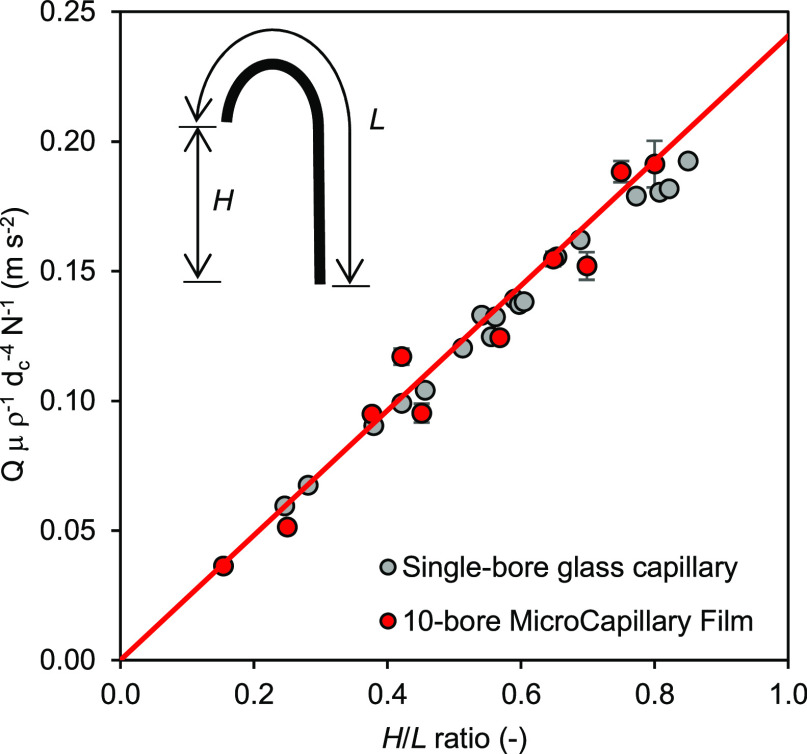
Normalized mean discharge flow rates from 10-bore MCF and single-bore glass capillary siphons at varying *H*/*L* ratios. The siphons were initially primed by dipping the head into Ultra-Pure water until all capillaries were filled and the bottom of the siphon submerged 2 mm below the liquid level that was kept constant. The head of the siphon was then dipped into a 150 μL droplet of DI water stained with 0.05% (w/w) indigo carmine, and the time taken to fully uptake the droplet was recorded. The neck distance was kept constant for the MCF siphons at precisely 19 mm (corresponding to a bending diameter of 11 mm), and the *L* of the strip varied in the range 40–100 mm. For the glass capillaries, *L* was kept constant at 142.4 mm and the *H*/*L* ratio varied by bending the glass capillary at different points along the length. As a simplification, it was assumed that the liquid height in the reagent wells and waste wells remained constant.

Through measuring the discharge flow rate of the siphon, *Q*, in a wide range of single-bore glass capillaries (with a measured mean internal diameter of 578 μm and mean contact angle of 50.9°; see the Supporting Information) and 10-bore MCF strips (with a measured mean hydraulic diameter *d_c_* = 204 ± 13.8 μm and mean contact angle of 67 ± 1.3°) with varying *H*/*L* ratios (range 0.15–0.85), we were able to demonstrate that the siphon concept is fully scalable (Figure 3), with flow rate, *Q*, solely governed by gravity, fluid properties, and the diameter of the porous channel following a simple pressure balance model (derivation provided in the Supporting Information):
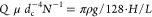
1where *N* is the number of parallel microcapillaries, μ is the kinematic fluid viscosity (0.001002 kg m^–1^ s^–1^ for water), *d*_c_ is mean hydraulic diameter of the microcapillary (or equivalent mean porous diameter for the membrane), ρ is the fluid density (998.2 kg m^–3^ for water), *g* is the acceleration due to gravity (9.81 m^2^ s^–1^), and *L* is the total length of the strip. Note that the model shown in eq 1 assumes a steady-state operation with the liquid head pressure and pressure drop resistance balanced and therefore is not suitable for predicting the instantaneous fluid velocity during, e.g., liquid priming or emptying of microcapillaries. The air–liquid meniscus presented during the priming of the siphon would add an extra force associated with surface tension that would need to be included in the pressure balance. Also, eq 1 assumes that the liquid head pressure remains constant during operation of the siphon, which was not the case in the siphon experiment shown in Figure 2. As can be seen in the bottom panel of Figure 2B, the discharge flow rate reduced slightly as the liquid level in the waste reservoir increased. The pressure balance that yielded eq 1 can also be easily adapted to consider time variations in the liquid pressure head, yet during operation of several siphons such as the ones shown in Figures 1C,D, we learned the importance of having a design that keeps the liquid head pressure approximately constant, which can be easily implemented using a shallow waste reservoir. This way, variations in the liquid head height remain insignificant compared to the liquid head pressure of the siphon.

Further experiments with MCF strips configured at varying neck bending diameters of 4, 7, and 11 mm and a fixed ratio *H*/*L* = 0.69 (results not shown) showed undetectable differences in resistance forces in the siphon neck, with a mean discharge flow rate of 3.63 ± 0.07 μL s^–1^.

### Siphon Effect in a Microporous Material

**Figure 4:**
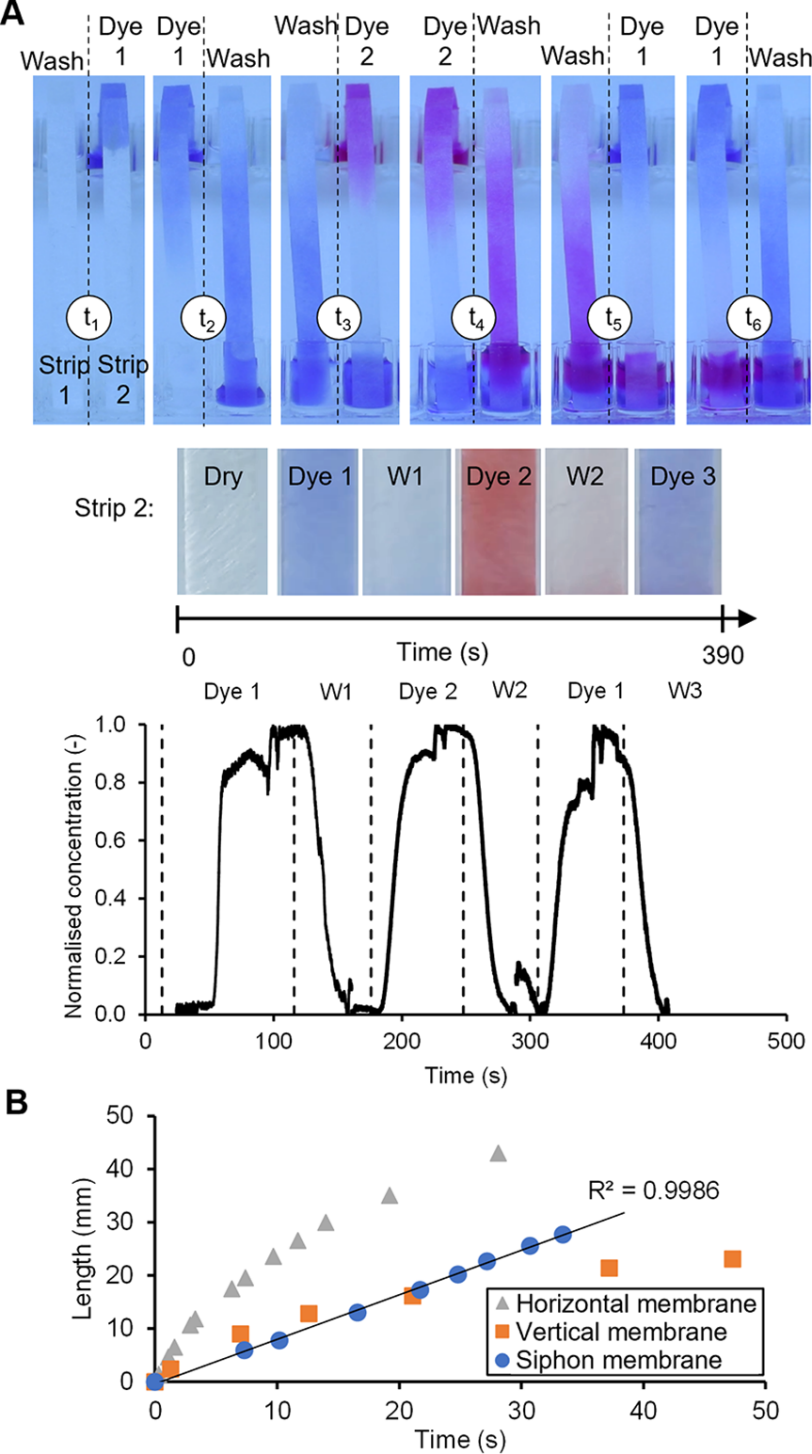
Operation of glass fiber porous matrix siphons. (A) Time snapshots (top) showing flow of dyed fluid (dye 1: bromophenol blue, 0.1 mg/mL; dye 2: phenol red, 0.1 mg/mL) in PBS-T, with the waste microwell emptied after each step in two parallel siphon membranes, designed with lateral flow conjugate pad, 7 mm wide, *L* = 80 mm, and *H* = 50 mm (more details provided in the methods section). Photographs of strip 2 (middle) at end of discharge of each reagent showing consistent and homogeneous concentration. Liquid breakthrough curves (bottom) with normalized concentration vs time for each reagent step through strip 1, as imaged at 36 mm from the outlet, with well-defined concentration plateaus for both the dyed fluid and washing buffer. (B) Position of the liquid meniscus tracked over time for a glass fiber membrane (*L* = 80 mm, width = 7 mm) held in vertical or horizontal position vs siphon design (same membrane as strip 1 shown in (A)), in PBS stained with indigo carmine (3 mg/mL), showing the movement of the fluid in the siphon format is linear in contrast to LFA membranes formatted in vertical or horizontal positions.

We demonstrated that the siphon effect is also achievable in strips of a porous substrate such as a porous glass fiber matrix used in LFA conjugate pads (Figure 4), with time scales comparable to the ones observed for the MCF siphons. A pair of strips imaged in Figure 4A (top) was operated in parallel but with an offset in reagent sequence to illustrate the dye and wash sequence. Breakthrough curves (bottom of Figure 4A) confirmed that the membrane siphon cycled through the full saturation of dye, to undetectable absorbance with small reagent volumes (200 μL for a 7 mm wide×80 mm long strip). Unlike the conventional LFA format with the horizontal fluid movement governed by a balance between capillary forces and resistance, the siphon membrane configuration is uniquely able to deliver constant fluid velocity at each step (Figure 4B). These results suggest that reconfiguration of the standard horizontal LFA into a siphon format (vertical, with a swan beak) could overcome both the uneven flow and varying flow resistance, acknowledged as two core limitations of LFAs. For multistep operation (Figure 4A), it enables combining continuous fluid flow (set by *H*) with stop–start flow features of the siphon, therefore offering a new level of fluidic control to LFA in porous materials.

In addition to enabling the use of a sequence of reagents and start–stop flow with a good level of control over the flow rate and incubation times (not possible in conventional LFAs), we further observed that operation of a porous membrane formatted according to the siphon design shown in Figure 1A, having a net *H* and a neck at a higher liquid height than the inlet, yielded a constant linear movement of the meniscus. This is not possible to see in the same membrane material operated in a vertical or horizontal format (Figure 4B), as in those cases fluid movement is dominated by resistance and/or capillary forces that vary as the fluid permeates the material.

### Application of MCF Siphons as Quantitative ELISA to Global Diagnostics

To use the microfluidic siphon for a high-performance POC device in a format suited to global healthcare needs, we developed 3D printed prototype cassettes (shown in Figure 1D) that enabled simultaneous operation of parallel siphon strips. The design can easily be mass-manufactured and has a 9 mm pitch compatible with conventional 96-well microplates and multichannel pipettes. Combined with the multibore MCF material, these stacked siphon holders offer high-throughput instrument-free testing, in addition to the multiplexing capabilities previously reported for this mass-manufactured material.^42^ Although microporous membranes remain a gold standard in POC testing, the two proof-of-concept ELISA applications presented here require quantitative capabilities that are difficult to realize with a porous matrix in an LFA. The optical transparency, multiplexing, and ″open fluidic″ features make the MCF strips especially fit for operation with siphon cassettes.

We demonstrated the clinical value of these siphon cassettes by carrying out a four-plex sandwich ELISA for serotype-specific quantitation of dengue NS1 protein with clinical human samples in Thailand and singleplex quantitation of SARS-CoV-2 IgM and IgG antibodies in simulated samples and convalescent COVID-19 human samples. Enzyme AttoPhos fluorescence signal was recorded with a smartphone camera and inexpensive LED illumination,^37,38^ allowing the near-patient or out-of-lab high-performance immunoassays needed for diagnostics and surveillance of important infections such as dengue and SARS-CoV-2.

**Figure 5:**
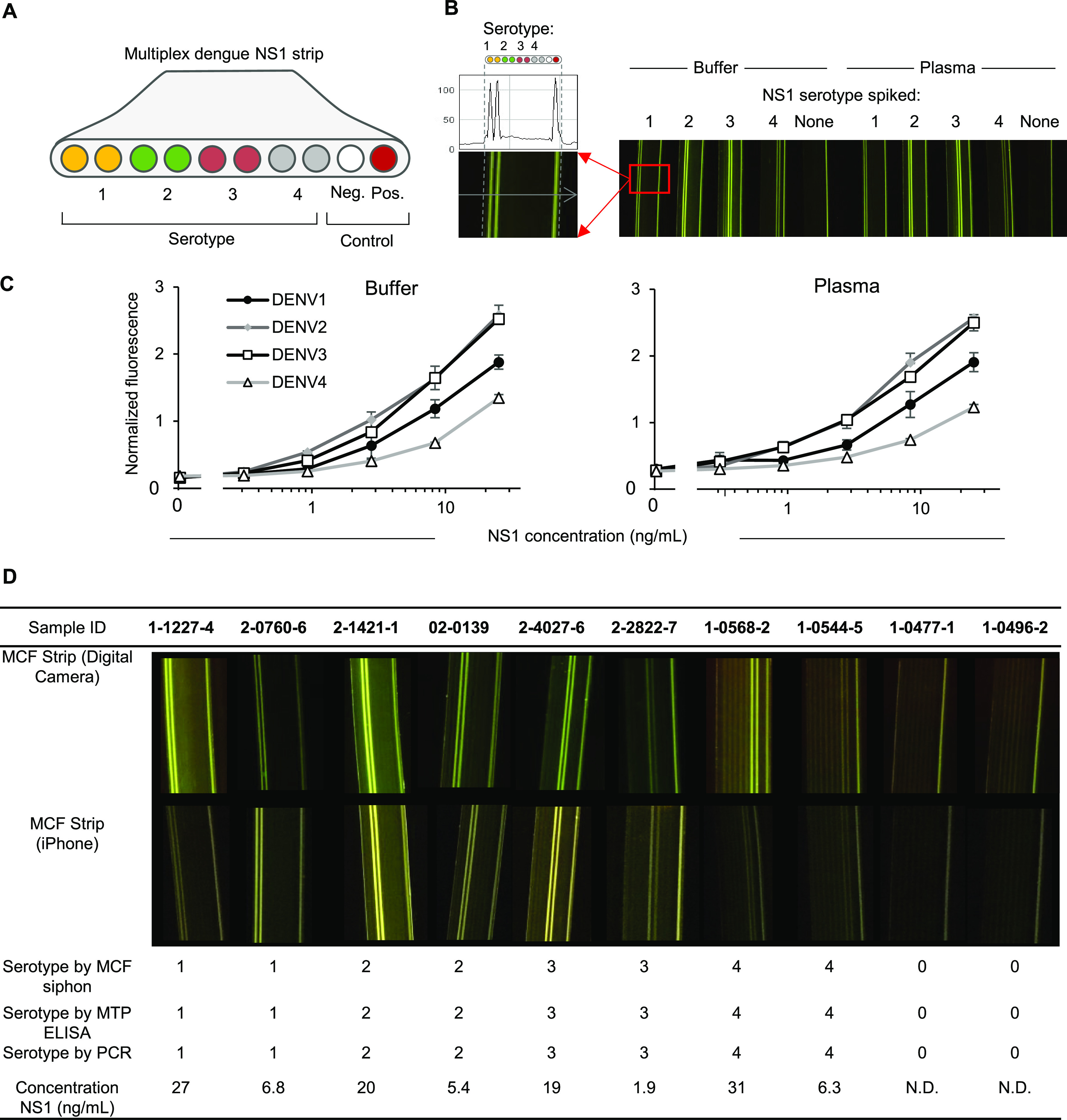
Enzyme-linked immunosorbent assay (ELISA) in MCF siphons enabling rapid smartphone diagnosis of dengue fever. (A) Configuration of serotype-specific NS1 capture antibodies and control capillaries within MCF test strips. (B) Image showing DENV NS1 quantitation spiked into buffer vs plasma, illustrating that only pairs of capillaries coated with capture antibodies corresponding to the NS1 serotype spiked show positive fluorescence. Profile plots across the MCF test strip (left-hand side) illustrate how fluorescence intensity was quantified for each individual capillary. (C) Quantitation of NS1 detection in plasma vs buffer for the four DENV serotypes. Mean fluorescence normalized to a fixed fluorescein reference sample was plotted from the duplicate capillaries, with error bars indicating 1 standard deviation. Data presented in (B) and (C) are representative of more than three independent standard curves in buffer vs plasma. (D) Digital camera and smartphone images of 10 multiplex DENV NS1 MCF tests using siphon fluidics of undiluted plasma samples compare two dengue fever patients for each of the four DENV serotypes vs two negative controls (*n* = 10). The serotype determined by PCR and NS1 serotype detected by siphon MCF were recorded below images, and the NS1 concentration indicated was calculated by comparison of fluorescent intensity in the patient sample to reference dilutions of recombinant NS1 as shown in (C).

Viral infections can be detected by measurement of antibodies against viral antigens (antibody tests) or of viral components directly (antigen tests). With 4 billion people at risk of dengue virus (DENV) infection and an estimated 100 million infections annually,^43,44^ access to decentralized diagnostic testing remains vital, and patients are typically tested first by LFAs that combine antibody and antigen detection; however, current point-of-care NS1 LFAs are not serotype-specific,^45^ so centralized testing remains essential for viral surveillance. We adapted a serotyping microplate ELISA that uses serotype-specific anti-NS1 antibody clones^46^ to siphon MCF devices. Multiplex capture strips with duplicate pairs of capillaries were coated internally through passive adsorption^40^ with serotype-selective capture antibodies (Table S1) against DENV NS1 (Figure 5A). Capture of viral antigen was detected using a pan-NS1 detection antibody. A positive control was included with a single capillary coated with recombinant NS1, alongside an uncoated negative control capillary. Only capillaries corresponding to a spiked recombinant NS1 showed positive fluorescence (Figure 5B), with full response curves showing clinically relevant sensitivity for all four serotypes (Figure 5C) with no difference in quantitation between the buffer and plasma. The four serotypes showed different analytical performance, with 1 ng/mL being readily detectable for serotypes 2 and 3, whereas serotypes 1 and 4 showed a weaker signal at the lower NS1 concentrations, yet all gave a strong signal at a 10 ng/mL threshold for patients with acute infection. Notably, the data obtained with siphon devices showed a limit of detection similar to that reported for serotyping NS1 microplate ELISA.^47^ Differences in analytical performance between serotypes are expected as the capture antibodies were selected primarily for serotype specificity and not for highest analytical sensitivity. A different set of serotype-specific anti-NS1 pairs showed a different pattern of analytical sensitivity in laboratory lateral flow devices, demonstrating the influence of antibody clone pairs on sensitivity.^48^ As dengue patients have from 10 ng/mL to 50 μg/mL NS1 in plasma during acute infection,^49^ the siphon MCF devices should be sufficiently sensitive for serotype-specific NS1 dengue diagnostics. PCR-serotyped pairs of patient samples from each serotype, plus matched negative controls (total *n* = 10), were tested using these devices, and NS1 was clearly detectable in each of them using both a digital camera and a smartphone (top two rows in Figure 5D). This demonstrated that multiplex viral antigen detection is feasible using siphon microfluidics imaged with a midrange smartphone (iPhone 6S). A large-scale clinical study of these devices with >250 samples found similar performance for the MCF siphon to microplate ELISA (manuscript submitted).

**Figure 6:**
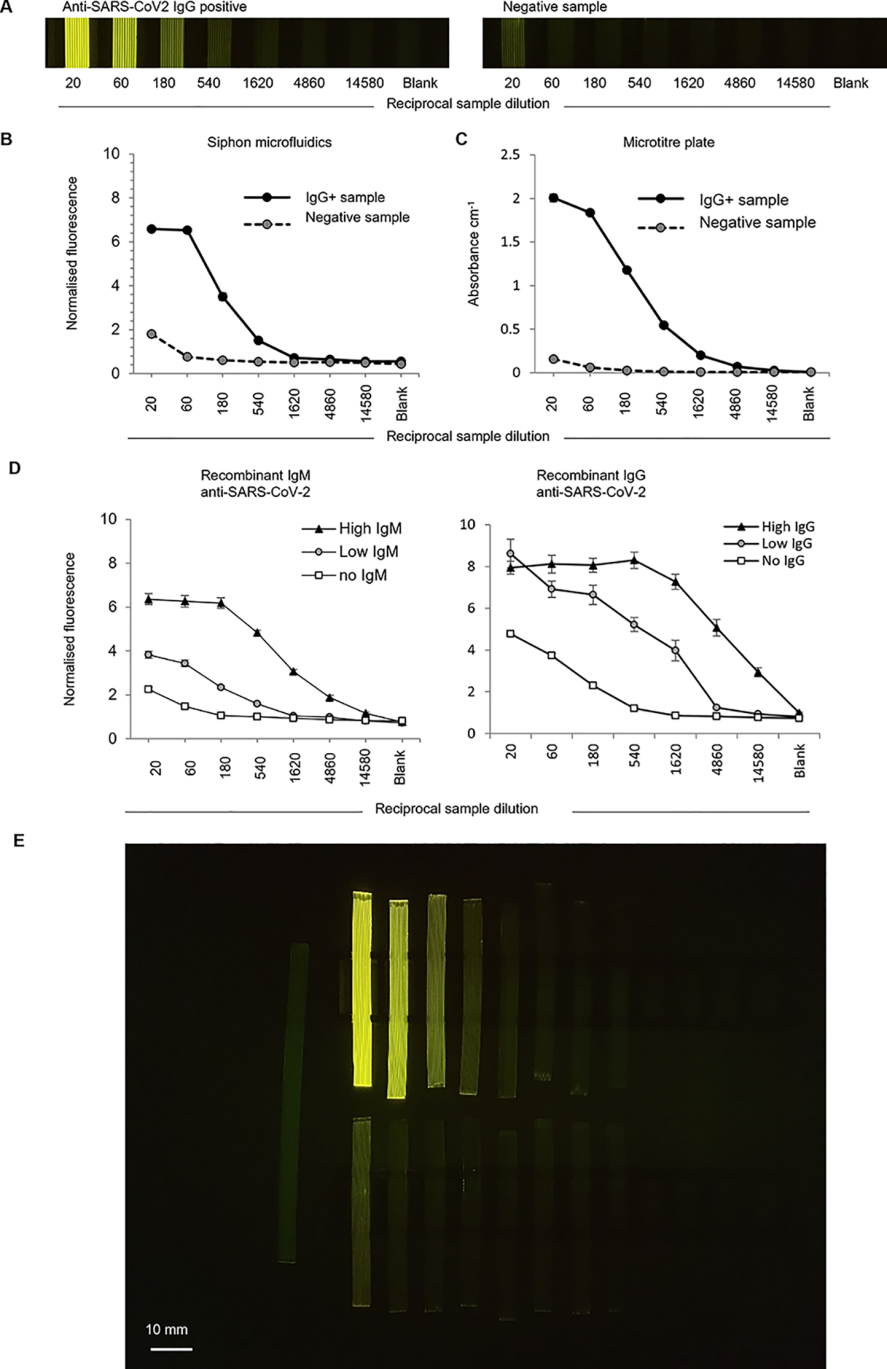
Smartphone enzyme-linked immunosorbent assay detection of IgM and IgG anti-viral antibodies to SARS-CoV-2 with MCF siphons in simulated acute vs post infection samples. (A) Detection of antiviral COVID-19 IgG antibodies in human plasma samples serially diluted and tested in a set of eight MCF siphon devices each coated with recombinant SARS-CoV2 spike protein within all 10 capillaries. (B) Comparison of quantitation by indirect ELISA of IgG antibodies against SARS-CoV2 spike protein in positive vs negative plasma sample in MCF siphon devices vs conventional microtiter plate, shown in (C) for each dilution the mean and standard deviation plotted represent 10 replicate MCF capillaries vs triplicate microtiter plate wells. Similar titration curves were seen in three replicate SARS-CoV2 indirect ELISA experiments. (D) Different levels of recombinant human IgM and IgG anti-SARS-CoV2 were spiked into prepandemic control serum to represent COVID-19 patients with low and high levels of IgM or IgG, respectively. These simulated patient samples were then serially diluted and tested using siphon MCF test strips coated with spike protein from SARS-CoV-2. (E) Smartphone imaging of 16 siphon MCF test strips quantifying IgG anti-viral antibody in seven serial dilutions of two human plasma samples. Top set is anti-SARS-CoV2 positive sample, with top dilution of 1:20 followed by six threefold serial dilutions and a blank. Lower row is the negative sample diluted and tested alongside the positive. A reference sample of fixed fluorescent dye concentration is visible to the left. Black MCF holders also visible. Images shown in (A) and fluorescence intensity values presented in (B) were taken in the middle section of the strips shown in (E), with no detectable change in greyscale pixel intensity found along the length of the strip. Note that error bars corresponding to 1 standard deviation were included in plots shown in (B), (C), and (D) yet in some cases were too small to be shown.

While viral antigens detected by sandwich immunoassay are valuable for some infections such as dengue, for other infections, anti-viral antibodies are more important. These can be detected using indirect ELISA by immobilizing viral antigens, incubating with the diluted sample, and probing for human immunoglobulins (e.g., IgG, IgM, or IgA). To quantify anti-viral antibody titer, a series of serial dilutions of the patient sample must be tested, with the end point titer reflecting the highest dilution at which a clear positive signal is retained. During many acute viral infections, the signal is retained beyond 10,000-fold dilution, illustrating the very high affinity binding characteristic of seroconversion. To establish quantitative indirect ELISA in siphon devices, MCF strips coated with the SARS-CoV-2 spike protein were tested with serial dilutions of recombinant IgM and IgG antibodies spiked into a prepandemic serum (Figure 6). After using these simulated samples to establish assay conditions that minimized assay background while allowing both high and low levels of IgG/M to be detected, we compared antibody titer quantitation in microplates vs siphon MCF devices (Figure 6B,C). Volunteer plasma samples previously identified as positive or negative for polyvalent anti-SARS-CoV-2 spike protein by conventional microplate ELISA^50^ were tested in parallel for IgG in siphon MCF vs microplates (Figure 6B,C).

We observed a clear difference between positive and negative samples (Figure 6A,D,E), with signal decaying in a similar pattern with dilution, indicating that siphon devices are suitable for serological COVID-19 testing including antibody titer measurement. Furthermore, the smartphone could record the full set of 16 × 10 microcapillary siphon devices comprising positive plus negative plasma samples, each serially diluted seven times, plus two negatives (i.e., 160 × 1 μL microcapillary data points) in a single photo (Figure 6E).

Note that both NS1 sandwich immunoassay and indirect SARS-CoV-2 ELISAs herein reported were carried out in a siphon cassette and MCF strips with a fixed *H*/*L* ratio. As the discharge flow rate of the siphon and superficial fluid velocity are linearly dependent on the *H*/*L* ratio, the reported ELISA performance should be carefully extrapolated to other *H*/*L* ratios through a more extensive validation. During this study, we noticed that the length of the siphon strip and washing volumes can impact on the signal-to-noise (and consequently performance) of bioassays; this will be the subject of future publications.

## Conclusions

Sequential siphon flow has been realized for a range of microbored/microporous material, relying entirely on gravity in contrast to all POC biosensing devices developed to date. Siphon devices can quickly be reconfigured with standard microplate ELISA reagents for singleplex or multiplex format to measure protein biomarkers, antigens, or antibodies without compromising analytical performance. This represents a major milestone in decentralizing high-performance diagnostic testing, removing the need of expensive automation, complex fluid handling, or skilled workforce. The siphon concept can transform POC testing with a new generation of high-performance power-free immunoassays transferring ELISA outside laboratories, with results digitally recorded by a smartphone camera to meet many rapidly changing global public health needs. Demonstration of improved analytical capabilities with porous membrane siphons and other *H*/*L* ratios is yet to be tested and will be the subject of future publications.

## Supporting Information Available

Brief description of contents: procedures for hydrophilic coating of MCF strips; fabrication of ″stacking″ immunoassay siphons; measurement of mean hydraulic diameter; equilibrium liquid height; colorimetric breakthrough in manual MCF and membrane siphons; colorimetric and fluorescent breakthrough curves in automated MCF siphon; loading of antigens/antibodies in MCF strips; derivation of pressure balance model for siphon flow; Figures S1, S2, and S3; and Tables S1 and S2 (PDF)
Compressed film showing time lapse operation of a four-channel MCF siphon cassette with a sequence of dyed liquid reagents (MP4)
Compressed film showing time lapse discharge of water stained with bromophenol blue from a four-channel MCF siphon cassette (top view) (MP4)
Compressed film showing time lapse operation of a one-channel MCF siphon cassette with a sequence of four reagents (MP4)
Compressed film showing perspective view of time lapse operation of a four-channel MCF siphon cassette, discharging washing buffer and DI water stained with red food dye (MP4)
Compressed film showing front view of time lapse operation of a four-channel MCF siphon cassette, involving priming of capillaries with DI water stained with bromophenol blue (MP4)
