# Supplementary material for: Gravity-Driven Microfluidic Siphons: Fluidic Characterization and Application to Quantitative Immunoassays
Source: ACS Sens. 2021 Dec 2;6(12):4338–48. doi: 10.1021/acssensors.1c01524 (PMC8728737; doi:10.1021/acssensors.1c01524)
Supplement: Supplementary file 1 — se1c01524_si_001.pdf [file se1c01524_si_001.pdf]

Supporting Information

Gravity-driven microfluidic siphons: fluidic characterization and application to quantitative immunoassays

Nuno M. Reis,[a],* Sarah H. Needs,[b] Sophie M. Jegouic,[a,b] Kirandeep K. Gill,[a] Sirintra Sirivisoot,[d] Scott Howard,[a] Jack Kempe,[a] Shaan Bola,[a] Kareem Al-Hakeem,[a] Ian M. Jones,[c] Tanapan Prommool,[e] Prasit Luangaram,[e] Panisadee Avirutnan,[d,e,f] Chunya Puttikhunt,[d,e,f] and Alexander D. Edwards[b]*

[a]Department of Chemical Engineering and Centre for Biosensors, Biodevices and Bioelectronics (C3Bio), University of Bath, Claverton Down, Bath BA2 7AY, United Kingdom, E-mail: n.m.reis@bath.ac.uk

[b]Reading School of Pharmacy, University of Reading, Whiteknights Campus, Reading, RG6 6AD United Kingdom, E-mail: a.d.edwards@reading.ac.uk

[c]School of Biological Sciences, University of Reading, Whiteknights Campus, Reading, RG6 6AJ, United Kingdom

[d]Dengue Hemorrhagic Fever Research Unit, Office for Research and Development, Faculty of Medicine Siriraj Hospital, Mahidol University, Bangkok, 10700, Thailand

[e]Molecular Biology of Dengue and Flaviviruses Research Team, Medical Molecular Biotechnology Research Group, National Center for Genetic Engineering and Biotechnology, National Science and Technology Development Agency, Pathum Thani, 73170, Thailand

[f]Siriraj Center of Research Excellence in Dengue and Emerging Pathogens
Faculty of Medicine Siriraj Hospital, Mahidol University, Bangkok, 10700, Thailand

*Corresponding authors: n.m.reis@bath.ac.uk, a.d.edwards@reading.ac.uk

Contents:

Supplementary Methods	S2
Figure S1	S3
Table S1	S6
Derivation of pressure balance model	S6
Supplementary Results	S7
Figure S2	S7
Figure S3	S8
Table S2	S9
Supplementary Video files	S10
Ethical statement	S11
Cited references	S11

SUPPLEMENTARY METHODS

Fluorescence Alkaline Phosphatase (AP) amplification with substrate, AttoPhos

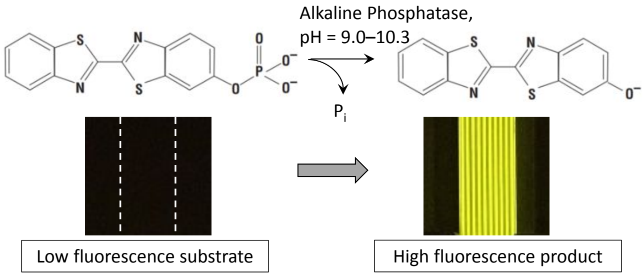

Figure S1. Reaction overview of AttoPhos conversion by AP. The substrate 2′-[2-benzothiazoyl]-6′-hydroxybenzothiazole phosphate (BBTP), with a low fluorescence, is cleaved by AP phosphatase to produce inorganic phosphate (Pi) and a highly-fluorescent alcohol, 2′-[2-benzothiazoyl]-6′-hydroxybenzothiazole (BBT).

Fabrication of 'stacking' immunoassay MCF siphons. A simple 3D printed cartridge was designed to hold *L*=47mm, *H*=25mm test strips in the siphon configuration, allowing the top inlet to interface with sample and reagent wells on a sample rack. The cartridge was stackable with push-fit pins, spaced with a 9mm pitch matching standard 96-well microplates (Figure 1D in main document). Cartridge, racks, and reagent wells were 3D printed from black poly-lactic acid filament (PLA-175-MIB-1000, Ooznest, Essex, UK) on a Prusa i3 Mk3 printer (Prusa Research, Prague, Czech Republic).

Measurement of mean hydraulic diameter in the MCF strips. The mean d_c for each microcapillary (Table S2 and Figure S2B) was computed from the ratio $d_c = 4A/P$, with the cross sectional area A and perimeter P measured in ImageJ (NIH, USA) from microphotographs of cross sections of the MCF taken with a AMG EVOS microscope (catalogue no: AMEFC4300, Thermo Fisher Scientific, Massachusetts, USA).

Hydrogel coating of 'plain' MCF siphons. A 20 mg ml^{-1} Polyvinyl Alcohol (PVOH) solution was prepared by slowly dissolving 4g of high-molecular weight PVOH (MW 146,000-186,000 g/mol, 99%+ hydrolysed, catalogue no: 363065, Sigma Aldrich, Dorset, UK) in 200mL of Ultra-Pure water in a calibrated glass container above a magnetic stirrer and hotplate at a temperature < 38°C. Then temperature was increased to 95°C for 30 minutes until all the PVOH had dissolved. As some evaporation occurred during the dissolution process, the volume of solution was adjusted by adding extra ultra-Pure water as required and 0.05% (w/w) Proclin 300 (catalogue no: 48912-U, sourced

from Sigma-Aldrich, Dorset, UK) added to prevent microbial growth. The hydrogel coating started by injecting 1.5 meter long MCF strips with the PVOH solution using a push-fit, PEEK luer connector developed in house[1] with a 25mL plastic syringe and left to incubate for 2 hours, after which the solution was blown out using a 25mL syringe filled with air. This was followed by gentle washings with Ultra-Pure water and 0.05% (w/w) Tween 20 (catalogue no: P1379, sourced from Sigma-Aldrich, Dorset, UK) at room temperature (20 ^{o}C) with a 25mL syringe, ensuring all capillaries were filled with liquid. Finally, all liquid was again withdrew from the microcapillaries and the MCF strip trimmed to produce shorter, 150mm long strips (or as required) that were dried again individually before storage in sample bag. The quality of the coating was tested by measuring the equilibrium liquid height in each individual microcapillary for every batch of MCF strips coated.

Equilibrium liquid height. For measuring the equilibrium liquid height in the coated MCF strips, the bottom end of a dried and clean glass capillary of MCF strip (at least 100 in length) was immersed in a transparent cuvette filled with Ultra-Pure water and the equilibrium position of the meniscus recorded as the vertical difference between the liquid level in the cuvette and the bottom of the meniscus in the capillary. This was repeated at least three times. Using the Laplace-Young equation,[2] the mean contact angle between the meniscus and the inner capillary surface were also calculated, being 67±1.3 degrees for the MCF (Table S2) and 50.9 degrees for the glass capillary. The measured mean equilibrium capillary rise was 32.5mm for the glass capillary and 58±3.0mm for the PVOH coated MCF strips (Table S2 and Figure S2C).

Colourimetric breakthrough curves with the MCF siphon strips. Indigo Carmine (85% dye content, catalogue no: 131164, Sigma-Aldrich, Dorset, UK), Bromophenol Blue (Sigma-Aldrich, Dorset, UK) or Phenol Red (Sigma-Aldrich, Dorset, UK) (with concentrations as indicated in text) was diluted in 10 mM PBS or 10 mM PBS-T. The strip was then illuminated with a white LED board (sourced from IO Rodeo Inc., Pasadena, USA) and imaged with a USB uEye XS industrial camera operated with dedicated software (IDS Imaging, Obersulm, Germany). Image analysis of absorbance values was carried out using ImageJ (National Institute of Health, USA).

Fluid breakthrough in glass fibre membranes. Fluid flow in membrane siphons was fully characterised using 200 µL of PBS with 0.05%Tween 20 (Sigma-Aldrich, Dorset, UK) wash buffer (PBS-T) alternated with 200 µL of 0.1 mg/mL Bromophenol Blue Sodium Salt (Thermo Fisher Scientific, UK) or Phenol Red Sodium Salt (Sigma Aldrich, UK) dye solution in PBS-T, with the waste microwell emptied after each step. The strip was then illuminated with a white LED board (sourced from IO Rodeo Inc., Pasadena, USA) and imaged with a USB uEye XS industrial camera

operated with dedicated software (IDS Imaging, Obersulm, Germany). This allowed to track the position of the fluid front vs time and length but also to determine the normalised concentration of fluid vs time (i.e. $F(t)$), based on absorbance measurement from the pixel greyscale. For comparison, we positioned additional glass fibre porous strips fabricated from the same material and with the same dimensions of the membrane siphon (grade 8964 lateral flow conjugate pad, Ahlstrom, Pont-Evêque, France; 7mm wide and 80mm long) in both vertical and horizontal positions and tracked the fluid front vs time and length during the priming of the siphon.

Absorbance measurements. Normalised breakthrough curves for membrane and microcapillary siphons involved image analysis using ImageJ (National Institute of Health, USA), consisting of splitting the channels from the RGB images and converting the greyscale pixel intensity in the resulting 8 bits images into absolute Absorbance values (Abs), by setting Abs = $-\log_{10}(I/I_0)$, where I is the mean pixel intensity in the strip/microcapillary and I_0 is the mean pixel intensity of the background. To make the results fully quantitative, both I and I_0 were kept in the 8-bit greyscale range of ~140-256. The normalised concentration was determined by normalising the Abs values between 0 and 1 for the plateaus with the washing buffer and dyed fluid, respectively. As each dyed fluid absorbed light on its own specific wavelength, the selection of the channel was based on maximum signal-to-noise ratio.

Loading of antigen/antibody coating into the MCF. Manufacturing of siphon immunoassay strips started with coating the inner surface of the Teflon FEP microcapillaries with the antigen/antibody. For that, reagents were loaded into the MCF strips (for singleplex SARS-CoV-2-S1 immunoassay) using a push-fit play PEEK luer connector for MCF developed in-house[36] fitted to a plastic syringe, and for multiplex DENV immunoassays, antibodies (Table S1) injected into individual capillaries with 1mL syringe fitted with 29G needle (BD Microlance, UK).

Colorimetric and fluorescent breakthrough curves in automated MCF siphon. A hydrophilic MCF strip (89mm in length) was fitted into the cassette shown in Figure 1C (see main document) and connected to a robotic arm consisting of a stepper motor mounted on a metallic shaft and able to move in both axial and vertical directions, whose horizontal and vertical movement and timings were pre-programmed with Microsoft Visual Studio 2015. The tip of the MCF was submerged in each well for 60s and alternated between 100µL reagent (indigo carmine for colorimetric, or fluorescein for fluorescent tracing) and 100µL Ultra-Pure water for washings. Fluorescent tracing used 0.5 mM diluted from 10 mg/mL fluorescein (catalogue no: 46955, sourced from Sigma-Aldrich, Dorset, UK) in anhydrous dimethyl sulfoxide (catalogue no: 276855, sourced from Sigma-Aldrich, Dorset, UK) stock solution and imaged with a USB uEye XS industrial camera operated with dedicated software (IDS Imaging, Obersulm, Germany) and excited with a blue 470 nm LED

board (IO Rodeo Inc., Pasadena, USA). Fluorescence signal for the strip was determined from the peak green channel pixel intensity using Image J (NIH, USA).

Table S1. Serotype specific DENV NS1 antibody clone pairs (all these antibody reagents were manufactured in house)[3]

Antibody clone	Use	Reactivity to DENV serotype	Type of epitope	Isotype
84B	Capture	DENV1	Conformation	IgG1
1B10	Capture	DENV2	Linear	IgG1
46A	Capture	DENV3	Conformation	IgG2a
4A	Capture	DENV4	Conformation	IgG2b
1F11	Detection	DENV1-4	Linear	IgG2a

Derivation of pressure balance model. Assuming steady-state operation and in the absence of an air-liquid interface inside the siphon (i.e. siphon fully-primed), fluid flow through a siphon microcapillary will experience two forces, being liquid pressure head (ΔP_H) and pressure drop ($\Delta P_{resistance}$):

$$\Delta P_{resistance} = \Delta P_H \qquad \text{(S1)}$$

As fluid flow is laminar, the term representing pressure drop through resistance can be replaced by the well-known Hagen–Poiseuille law:

$$\Delta P_{resistance} = 128\ \mu\, Q\, L\ \pi^{-1}\ \mathrm{d_c}^{-4} \qquad \text{(S2)}$$

For a circular microcapillary with inner diameter d_c, total length L, and configured as a 'beak swan' siphon with a net hydrostatic liquid head, H, the pressure balance can be re-written as:

$$128\ \mu\, Q\, L\ \pi^{-1}\ \mathrm{d_c}^{-4} = \rho\, g\, H \qquad \text{(S3)}$$

To yield flow rate Q discharged, evenly, through N parallel microcapillaries, Eq. (S3) can be re-written as:

$$128\ \mu\, Q\, N^{-1}\, L\ \pi^{-1}\ \mathrm{d_c}^{-4} = \rho\, g\, H \qquad \text{(S4)}$$

Finally, Eq. (S4) can be rearranged as a function of H/L ratio and fluid properties yielding the total discharge flow rate for the siphon:

$$128\ Q\, \mu\, N^{-1}\ \mathrm{d_c}^{-4} = \pi\ \rho\, g/128\ \cdot H/L \qquad \text{(S4)}$$

SUPPLEMENTARY RESULTS

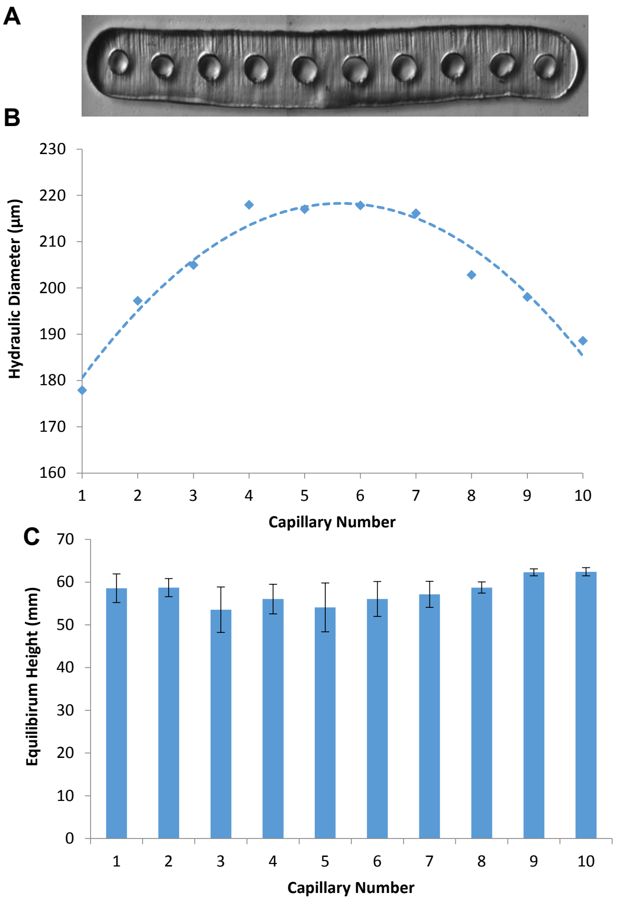

Figure S2. PVOH coated MCF strip material used. A) Microphotograph of the cross section of FEP Teflon MCF strip with 10 capillaries**.** B) Mean hydraulic diameter (replicates from Table S2). Each data point represents the average of 3 independent measurements. C) Equilibrium capillary height measure for each individual microcapillary. Error bars represent ±1 standard deviation from 10 independent measurements.

Breakthrough curves showed capability of the siphon platform to operate with full saturation for tracing and washing and high reproducibility in both absorbance (colourimetric) and fluorescence imaging setups (Figure S3).

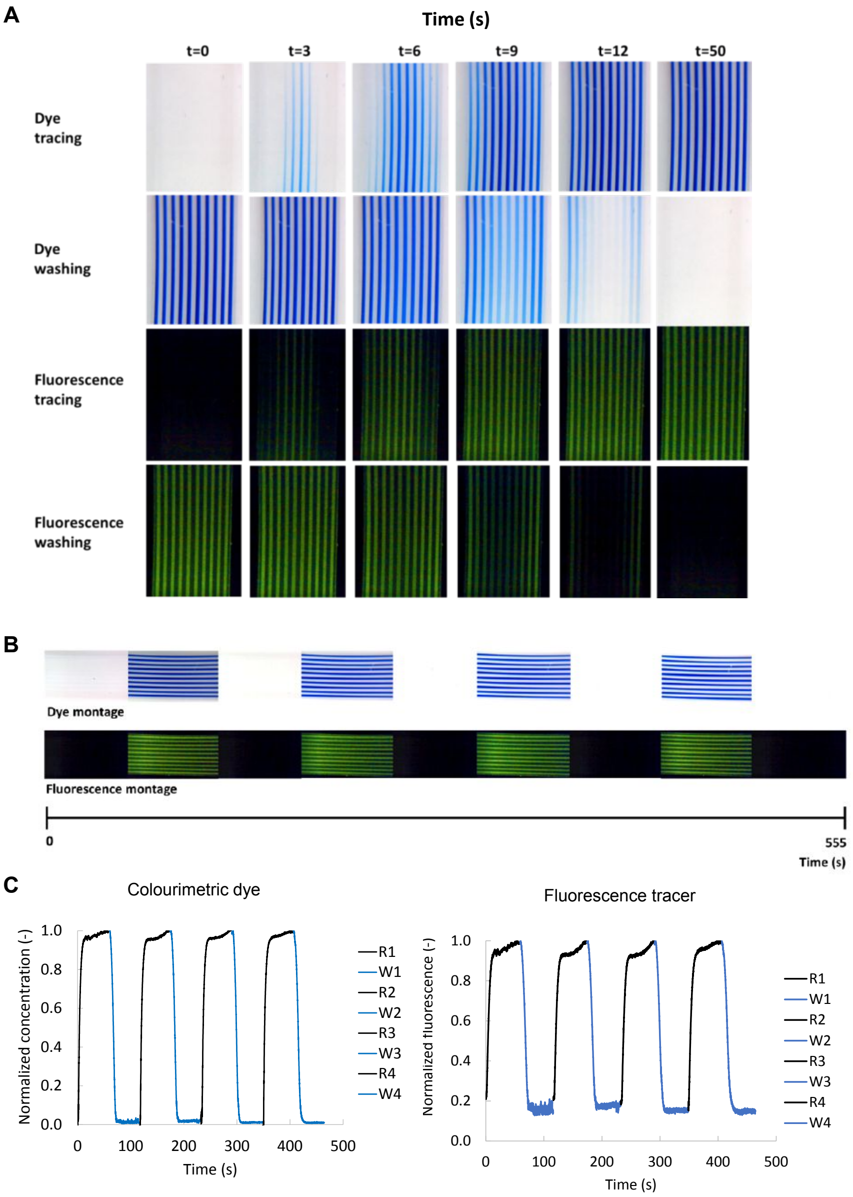

Figure S3. Fluid flow in an automated siphon cassette in colourimetric and fluorescence imaging modes. A) Microphotographs showing reagents are rapidly homogenised in individual capillaries through simple molecular diffusion, during aspiration and washing (only one aspiration and one washing is shown in this time sequence). Results were very consistent with fluid traced with coloured dye (3 mg/ml of Indigo carmine) or fluorescein (0.5 mM). B) Montage of image of the strip during multiple aspiration of washings, showing excellent qualitative breakthrough of fluid. C) Normalised concentration and fluorescence plots showing quantitatively aspirations and washings are readily reproducible.

Table S2. Inner dimension and mean equilibrium height (measured) and mean contact angle (estimated) for the PVOH coated MCF material. Mean contact angle was estimated based on inner *a* and *b* dimensions and mean equilibrium height with the modified Laplace-Young equation presented elsewhere[2]

**Capillary Number**	***A*** (μm^2)	***P*** (μm)	**Major Axis, a** (μm)	**Minor Axis, b** (μm)	d_c (μm)	**Mean Equilibrium Height** (mm)	**Mean Contact Angle** (°)
1	24876	560.3	182.2	173.8	177.6	58.5	69.5
2	30553	620.0	197.7	196.8	197.1	58.7	67.1
3	33032	644.3	212.6	197.9	205.1	53.5	68.4
4	37318	686.2	218.0	218.0	217.5	56.0	65.8
5	36994	681.9	217.1	217.0	217.0	54.1	66.8
6	37433	687.7	233.1	204.5	217.7	56.0	65.8
7	36697	678.7	216.3	216.1	216.3	57.1	65.5
8	32353	639.0	210.4	195.8	202.5	58.7	66.4
9	31422	637.5	230.4	173.7	197.2	62.3	65.5
10	27940	591.7	188.8	188.5	188.9	62.4	66.7

SUPPLEMENTARY VIDEO FILES

File name: Operation_of_4_Channel_MCF_siphon_cassette.mp4

Brief description of contents: Compressed film showing time lapse operation of a 4-channel MCF siphon cassette with a sequence of dyed liquid reagents.

File name: Reagent _removal.mp4

Brief description of contents: Compressed film showing time lapse discharge of water stained with bromophenol blue from a 4-channel MCF siphon cassette (top view).

File name: Reagent _colour_change.mp4

Brief description of contents: Compressed film showing time lapse operation of a 1-channel MCF siphon cassette with a sequence of four reagents.

File name: Changing_reagent.mp4

Brief description of contents: Compressed film showing perspective view of time lapse operation of a 4-channel MCF siphon cassette, discharging washing buffer and DI waster stained with red food dye.

File name: Adding_first_sample.mp4

Brief description of contents: Compressed film showing front view of time lapse operation of a 4-channel MCF siphon cassette, involving priming of capillaries with DI water stained with bromophenol blue.

Ethical statement

This study was approved by the ethics committee of Siriraj Institutional Review Board, Faculty of Medicine Siriraj Hospital, Mahidol University, Thailand. The written formal consent was obtained from parents or guardians before enrolment of each patient.

Cited References

[1] Alves, I. P.; Reis, N. M. Microfluidic Smartphone Quantitation of Escherichia Coli in Synthetic Urine. *Biosens. Bioelectron.* **2019**. https://doi.org/10.1016/j.bios.2019.111624.I. P.

[2] Reis, N. M.; Pivetal, J.; Loo-Zazueta, A. L.; Barros, J.; Edwards, A. D. Lab on a Stick: Multi-Analyte Cellular Assays in a Microfluidic Dipstick. *Lab Chip* **2016**, *16*, 2891–2899. https://doi.org/10.1039/C6LC00332J.

[3] Puttikhunt, C.; Prommool, T.; U-thainual, N.; Ong-ajchaowlerd, P.; Yoosook, K.; Tawilert, C.; Duangchinda, T.; Jairangsri, A.; Tangthawornchaikul, N.; Malasit, P.; Kasinrerk, W. The Development of a Novel Serotyping-NS1-ELISA to Identify Serotypes of Dengue Virus. *J. Clin. Virol.* **2011**. https://doi.org/10.1016/j.jcv.2011.01.001.
